# Fracture of the medial intercondylar eminence of the tibia in horses treated by arthroscopic fragment removal (21 horses)

**DOI:** 10.1111/evj.12720

**Published:** 2017-08-15

**Authors:** L. M. Rubio‐Martínez, W. R. Redding, B. Bladon, H. Wilderjans, R. J. Payne, C. Tessier, O. Geffroy, R. Parker, C. Bell, F. A. Collingwood

**Affiliations:** ^1^ Philip Leverhulme Equine Hospital Institute of Veterinary Science University of Liverpool Neston Wirral UK; ^2^ North Carolina State University College of Veterinary Medicine Raleigh North Carolina USA; ^3^ Donnington Grove Veterinary Group Newbury Berkshire UK; ^4^ Dierenkliniek De Bosdreef Moerbeke‐Waas Belgium; ^5^ Rossdales Equine Hospital and Diagnostic Centre Exning, Newmarket Suffolk UK; ^6^ ONIRIS Chirurgie et anesthésie Nantes France; ^7^ Liphook Equine Hospital Liphook Hampshire UK; ^8^ Elders Equine Veterinary Service Winnipeg Manitoba Canada

**Keywords:** horse, fracture, stifle, intercondylar eminence of the tibia, arthroscopy

## Abstract

**Background:**

Fractures of the medial intercondylar eminence of the tibia (MICET) are scarcely reported in horses.

**Objectives:**

To report the clinical and diagnostic findings, surgical treatment and outcome in a series of horses presented with MICET fracture and treated with arthroscopic fragment removal.

**Study design:**

Multicentre retrospective case series.

**Methods:**

Case records of horses diagnosed with MICET fractures that had undergone surgical treatment were reviewed. Follow‐up information was obtained from re‐examination visits and/or owners.

**Results:**

Twenty‐one cases were identified at 9 equine hospitals between 2004 and 2016. A history of trauma and acute onset of lameness was reported in 12 horses. All cases underwent fracture removal via arthroscopy of the medial femorotibial joint. The cranial cruciate ligament was intact in 6 horses and damaged in 15 horses (damage was ≤25% [n = 9], 25–50% [n = 4] or ≥50% [n = 2] of the cross‐sectional area). The cranial ligament of the medial meniscus was damaged in 11 horses (≤25% [n = 8], 25–50% [n = 3]). The medial meniscus was damaged in 5 horses and articular cartilage damage was identified in 14 horses (mild [n = 8], moderate [n = 6]). Follow‐up information (median 14 months; 4 months–6 years) was available for 20 cases; 2 horses were sound but convalescing; 13 horses were sound and returned to their previous or expected use. Of the 4 horses with the most severe changes to the articular soft tissue structures, 2 remained lame and 2 were subjected to euthanasia because of persistent lameness.

**Main limitations:**

The retrospective, multicentre nature of this study and the limited number of horses are the main limitations.

**Conclusions:**

Fractures of the MICET are commonly associated with a traumatic event. Prompt diagnosis and early arthroscopic removal of the fracture are recommended.

## Introduction

The intercondylar eminences of the tibia comprise of 2 bone prominences that increase the congruity of the articular surfaces in the axial aspect of the tibial and femoral condyles 1. The medial eminence is larger than the lateral eminence and its proximal aspect is in close apposition to the axial aspect of the medial femoral condyle. Although relatively uncommon and scarcely reported in the literature, fractures at this location primarily involve the medial intercondylar eminence of the tibia (MICET) 2, 3, 4.

These fractures were first described as avulsion fractures of the distal attachment of the cranial cruciate ligaments (CraCL) based on MICET fractures being commonly observed in cases with CraCL damage 2, 3, 5, 6. However, the CraCL distal attachment site is cranial and immediately lateral to the MICET and, therefore, these fractures are no longer considered avulsion fractures but probably caused by trauma from a lateral force from the medial femoral condyle on the intercondylar eminence 4, 7. This is supported by anecdotal descriptions of MICET fractures associated with minimal or no damage to the CraCL 7. Associated damage to other articular structures such as articular cartilage, menisci, meniscotibial ligaments and cruciate ligaments seem to vary among cases and may be associated with outcome 7, 8.

Although internal fixation has been reported in one case 4, arthroscopic treatment is currently the treatment of choice for affected animals 7. However, to the authors' knowledge, full description of clinical and diagnostic findings in affected animals and results of arthroscopic treatment are limited to single case reports 2, 4, 5, 9. The objective of this study was to report the clinical and diagnostic findings, surgical treatment and outcome in a series of horses presented with MICET fracture.

## Materials and methods

### Study population

Horses that were diagnosed with fracture of the MICET and underwent arthroscopic fragment removal at 9 equine hospitals (Philip Leverhulme Equine Hospital, University of Liverpool, UK; North Caroline State University, USA; Donnington Grove Veterinary Group, UK; Dierenkliniek De Bosdreef Moerbeke‐Waas, Belgium; Rossdales Equine Hospital and Diagnostic Centre, UK; Oniris‐Nantes, France; Vet‐Suisse‐Faculty of the University of Bern, Switzerland; Liphook Equine Hospital, UK; Elders Equine Veterinary Service, Canada) were included in the study.

### Data collection

Retrospective analysis of case records was performed. Data collected for each horse included age, breed, sex, previous or intended use of the horse, reason for referral, history of trauma and duration of lameness before presentation, clinical findings, evidence of lameness as per subjective assessment by the attending clinician, lame limb and subjective lameness grade 10. Use of intra‐articular diagnostic anaesthesia of the affected joint and clinical response to that was recorded. The following data on the imaging diagnostic investigations were retrieved: number and name of radiographic projections, visualisation of fracture on the radiographic images; number and size of fragments; other radiographic findings; any evidence of the fracture observed ultrasonographically; and other findings on ultrasonographic examination. Arthroscopic examination of the affected and contralateral stifle joints and arthroscopic approaches used were reviewed. Veterinary surgeons involved in the management of the cases were asked which radiographic projection they considered most useful for identifying the fractures and which arthroscopic approach they preferred. They were also asked to grade the damage to the CraCL, cranial ligament of the medial meniscus, medial meniscus and articular cartilage of the medial femoral condyle. Damage to these structures was subjectively graded by the individual surgeon who performed the arthroscopic examination. Damage of articular cartilage was graded as mild (few superficial wear lines), moderate (deep but not full thickness wear lines or areas with partial thickness loss of articular cartilage) or severe (full thickness loss of articular cartilage and subchondral bone exposure). Damage to the individual ligaments was recorded as estimated % of cross‐section area of the ligament involved. Meniscal tears were graded as previously reported 7.

Intraoperative and post‐operative treatments and post‐operative management and rehabilitation programmes were reviewed. Follow‐up information was obtained from clinical records obtained at the time of re‐examinations at the hospital or by telephone conversations with referring veterinarians, owners, trainers and/or online race records. Information retrieved included horse's survival, cause of death if horse was no longer alive, total convalescence time, persistent lameness at the end of the convalescence period, whether lameness was associated with the original stifle injury in cases with persistent lameness, horse's return to previous or intended use and reason for change of use if applicable.

### Data analysis

Data are presented descriptively with median and ranges when appropriate.

## Results

### Case details and clinical signs

Twenty‐one cases were identified between January 2004 and March 2016. The median age of horses was 10 years (range: 1–18 years), 13 males, 8 females. Case background and horse use are presented in Supplementary Item 1. Reasons for referral were lameness investigation (n = 11) and surgery (n = 10). The median duration of lameness was 31 days (range: 1–90 days). History of trauma associated with an acute onset of lameness was reported in 12 horses (5 collisions, 4 falls, 5 cast in the stable, one road accident). The right stifle was affected in 11 cases and the left stifle in 10 cases. At the time of initial examination all horses displayed lameness in the affected limb (Supplementary Item 1). Effusion of the medial femorotibial joint (MFTJ) and femoropatellar joint was present in 18 horses.

### Diagnostic procedures

**Figure 1:**
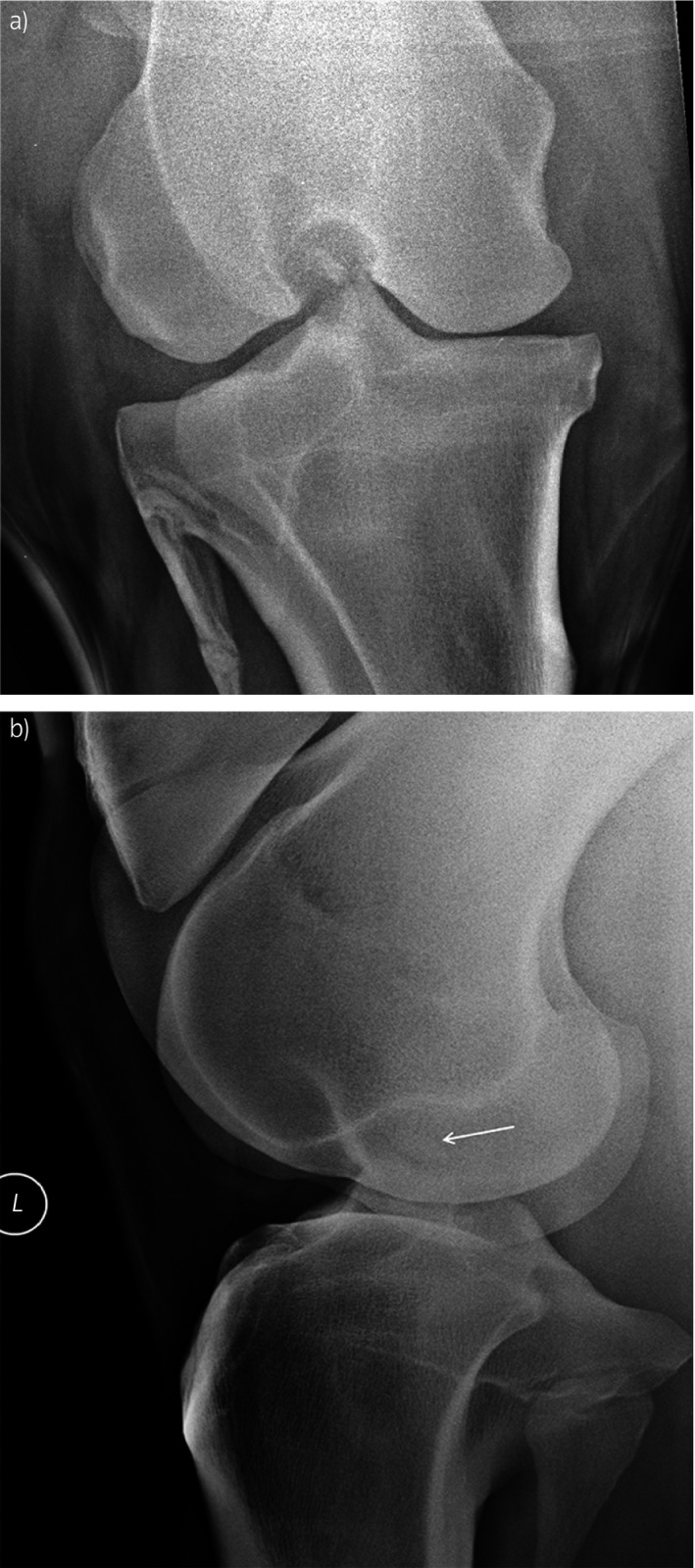
Caudocranial a) and lateromedial b) radiographic views of the stifle of a horse with a displaced fracture of the medial intercondylar eminence of the tibia. The fractured medial intercondylar eminence of the tibia is indicated with an arrow in b).

Intra‐articular analgesia was used in 9 cases and yielded a marked improvement (>50%) in lameness in all except one case where the improvement was graded as 40%. Radiographic examination of the affected stifle was performed in all cases and fracture of the MICET was identified radiographically in 19 cases (Fig 1 and Supplementary Item 2). In the other 2 cases, the radiographic images were of poor quality (Case 16) and showed collapse of the MFTJ (Case 18), which obscured the identification of fragments. Other radiographic findings in combination with the fracture were observed in 8 horses including osteophystosis in 5 horses and, in one case each: focal mineralisation of the medial meniscus, MFTJ collapse, flattening of the medial femoral condyle and a proximal avulsion fracture associated with the middle patellar ligament (Supplementary Item 2). The attending veterinary surgeon on the cases considered that the caudocranial view was most useful for identifying the MICET fracture in 14 cases, the flexed lateromedial in 2 cases and either the caudocranial or lateromedial views in 3 cases. Ultrasonographic examination of the affected stifle was performed in 15 cases and revealed synovial effusion of the MFTJ in all cases. The fragments were identified in 9 cases and presence of soft tissue abnormalities other than synovial effusion of the MFTJ in 8 cases including disruption of the medial collateral ligament of the MFTJ, lesions associated with the medial meniscus (5 cases), medial collateral ligament of the MFTJ (2 horses), cranial meniscotibial ligament (one horse), middle patellar ligament (one horse; Supplementary Item 2).

### Surgical approach

All cases underwent arthroscopic examination of the affected MFTJ joint and arthroscopic removal of the fractured MICET by a total of 9 surgeons (Supplementary Item 3). The lateral arthroscopic approach to the MFTJ 7 was most commonly used (Supplementary Item 4). A cranial arthroscopic approach 7 was used in 3 cases and the approach from the femoropatellar joint 7 into the MFTJ was used in 5 cases. In Case 3, a lateral arthroscopic approach was impeded by a cranially displaced MICET fracture and a cranial arthroscopic approach was then used. No other technical complications were recorded. In all cases a cranial instrument portal was used 7. Of the 9 surgeons involved, 5 would prefer the lateral arthroscopic approach, one would select a cranial approach and 3 would use the approach from the femoropatellar joint.

Number and size of fragments are included in Supplementary Item 2. Integrity or damage to the different intra‐synovial structures is included in Supplementary Item 4. Adhesion between the medial aspect of the CraCL and the fractured MICET requiring sharp dissection between both structures was reported in 12 cases. Removal of the fragments was accomplished by the use of a motorised resector and sharp debridement (18 cases), and/or a radiofrequency probe (6 cases). Autologous conditioned serum was injected into the cranial medial meniscotibial ligament intraoperatively in Cases 20 and 21.

The lateral femorotibial joint was examined in 18 cases and mild intra‐articular injuries were recorded in 6 horses (Supplementary Item 4). The femoropatellar joint was examined in 4 horses and no clinically relevant abnormalities were observed. The contralateral stifle joint was examined arthroscopically in 2 cases as requested by the referring veterinary surgeon or because of bilateral lameness. In both cases, mild articular cartilage fibrillation was evident on the medial femoral condyle.

Post‐operative radiographs revealed the presence of remaining fragments in 6 cases. Cases 4, 8, 13 had one fragment and Case 19 had two fragments remaining. A second arthroscopy for removal of the remaining fragment was performed in Case 8. In the other 3 horses, fragments were left in place because of their small size, being embedded in soft tissue or not visible arthroscopically. These 4 horses achieved soundness and returned to their previous use. Post‐operative radiographs in Cases 12 and 14 revealed the presence of 3 and 4 remaining fragments, respectively. These 2 horses did not have a second arthroscopic surgery and did not achieve soundness.

### Post‐operative care

Post‐operative care included variable periods of stable rest and in‐hand walking exercise (4–12 weeks) followed by paddock rest (6–20 weeks). Horses with shorter periods of stable rest tended to have longer periods of paddock turn‐out. This was followed by a gradual introduction into exercise. During the rehabilitation period, a horse‐walker was used in 3 horses, and a treadmill was used in 3 horses. Post‐operative intra‐articular medication included triamcinolone (Cases 2, 6, 8, 13, 16), betamethasone (Cases 17, 18, 19), methylprednisolone (Case 9), hyaluronic acid (Cases 2, 5, 9, 13, 16, 17, 18, 19, 20, 21), IRAP (Cases 14, 20, 21) and bone derived mesenchymal stem cells (Case 14) at variable time periods after surgery. Polysulfated glycosaminoglycans and chondroitin sulfate were administered orally to horse 18. Individual details on total convalescence time, follow‐up information and return to previous use are included in Supplementary Item 5. Overall, from the 20 cases with follow‐up, 16 horses (76.19%) were sound: 13 horses had returned to their previous or expected use (65%), 3 horses were still convalescing and one horse previously used for polo was now being used for general purpose as per owner's decision. Two horses (Cases 12 and 14) remained lame; one was used as a broodmare and the other had been retired. Two horses (Cases 3 and 4) had been subjected to euthanasia because of persistent lameness. These last 4 horses had the most severe changes to the articular soft tissue structures of the MFTJ. Post‐mortem computed tomography examination of the affected stifle of horse 4, 6 days after arthroscopic surgery, revealed severe damage to the subchondral bone of femoral and tibial condyles including osseous cyst‐like lesions and bone lesions at the distal attachment sites of the CraCL that were not evident radiographically. This horse was graded as the one with the most severe soft tissue damage in the joint and had the largest MICET fracture.

## Discussion

This report indicates that fractures of the MICET are commonly associated with a traumatic event and affected horses present with variable degrees of lameness and damage to articular soft tissue structures. Synovial effusion and lameness are the most frequently observed clinical signs. Intra‐articular anaesthesia of the MFTJ was commonly associated with significant improvement in the degree of lameness.

A lateral force from the medial condyle against the opposing MICET has been suggested as the cause of MICET fracture 4 and a traumatic incident was reported in 78% of the horses included in this study. The MICET is in close apposition to the axial aspect of the medial femoral condyle and seems to be actively involved in joint loading as the opposing area in the medial femoral condyle has high subchondral bone density 11, 12, 13. Lateral motion of the medial femoral condyle during the traumatic episode could contribute to the fracture 4, which could also be augmented by the outward rotation of the tibia that occurs during extension of the stifle joint 14. This study also confirms that MICET fractures are not avulsion fractures of the distal attachment of the CraCL as the CraCL was intact in 28.5% and only mildly damaged in 42.8% of the horses. This is further supported by an experimental study where the equine CraCL subjected to tension failed at mid‐substance in all specimens, avulsion fractures of the distal attachment of the CraCL without mid‐substance ligament rupture were not observed, and combined mid‐substance and fracture failure occurred in 4 of the 14 specimens tested 15.

Despite these observations, damage to the CraCL may be also present in some horses with MICET fracture. The CraCL does not attach to the MICET, but both structures are in close anatomical proximity as the distal attachment site of the CraCL lies immediately lateral and cranial to the MICET 1. In fact, sharp dissection between the CraCL and the lateral and cranial margins of the MICET was required in 12 cases (57.14%), including some with an intact CraCL. Iatrogenic damage to intact CraCL during removal of the MICET fragment has also been reported 4. Therefore, although MICET fractures are not avulsion fractures, damage to the CraCL may be present because of their anatomical proximity. In an experimental setting, combined mid‐substance CraCL failure and fracture of the MICET was suggested to occur more frequently in old animals 15. Although this injury combination was observed in some of the younger horses included in this study, further studies are necessary to further investigate this.

Soft tissue structures other than the CraCL were less frequently involved. These injuries may result from the initial traumatic event or develop as a consequence of the joint instability that is likely to develop as both MICET and associated soft tissues are fundamental in maintaining stability of the stifle joint 13, 15. The cranial ligament of the medial meniscus is located directly cranial to the MICET 16 and within the articular region subject to greatest compressive strain during stifle extension 17. These reasons may explain why the cranial ligament of the medial meniscus was the second most frequently injured after the CraCL in horses with MICET fractures. Damage, however, was mild. Damage to the articular cartilage of the medial femoral condyle was present in 66% of horses, and was mild in half of them. The close apposition between the articular surface of the MICET and the axial aspect of the medial femoral condyle may explain articular cartilage damage resulting from the opposing fracture margins, especially as the fracture was displaced in most of the cases 4. Presence of damage in the lateral femorotibial or contralateral stifle was uncommon.

Radiographic examination revealed the presence of MICET fracture in most cases and the caudocranial view was selected by most of the surgeons as the most useful radiographic view that allowed identification of the fracture. The fracture was not identified radiographically in 2 cases, which could be associated with suboptimal image quality in the intercondylar area of the stifle 18. The flexed lateromedial radiographic view was used in 50% of the cases and was considered most useful to identify the presence of MICET fracture in some cases. The flexed lateromedial radiographic projection is useful to assess the MICET area, especially in cases with meniscal tears that are frequently associated with new bone formation on the MICET 18 but this was not reported in the MICET fracture cases included in this study.

Ultrasonography allowed identification of the fracture in most and soft tissue injuries in approximately half of the cases on which this diagnostic technique was used. Ultrasonography is very useful to identify soft tissue damage and especially meniscal and patellar ligament injuries, which may not be visualised arthroscopically 18, 19, 20. More advanced imaging modalities, such as computed tomography or magnetic resonance, provide a more complete examination of the different structures of this complicated joint including soft tissue, articular cartilage and subchondral bone that cannot be accurately assessed by more conventional diagnostic imaging modalities 21, 22, 23. This occurred with Case 4 in our study, whereby severe subchondral bone degeneration was not diagnosed on routine plain radiographs but was only visible on post‐mortem computed tomographic examination.

A lateral arthroscopic approach to the cranial compartment of the MFTJ was used most frequently. While a cranial arthroscopic examination was used less frequently, it provides a more consistent examination of the intercondylar area, which is the area of interest in the horses with MICET fractures. Triangulation capability with the instrument, however, may be limited with a cranial approach. The approach to the cranial pouch of the MFTJ from the femoropatellar joint was used in 5 horses. The use of this approach has been reported with lower frequency in the literature but it allows access to both cranial compartments of the MFTJ and lateral femorotibial joint (FTJ). It provides good visualisation of the axial aspect of the femorotibial joints while leaving a clear area cranially for instrument placement which may be useful in these cases 7. Examination of the cranial compartment of the lateral FTJ was performed in most cases and the presence of pathology was uncommon. Examination of the caudal compartments of either the medial or lateral FTJs was not performed in any of the cases and, therefore, injuries in those locations may have been missed. Technical difficulties with arthroscope and instrument placement were reported in Case 3. This horse had a large fragment that was cranially displaced and interfered with the placement of the arthroscopic portal following the lateral approach while a cranial approach allowed access to the joint. Preoperative assessment of the location of displaced MICET fragments by radiography or ultrasonography is recommended. Although all of these cases were treated with arthroscopic removal of the MICET fractures, internal fixation has been reported in one case but was associated with technical difficulties 4.

In this case series MICET fractures were associated with a good prognosis. Of the horses for which follow‐up was available, 76.19% were reported as sound and 65% had returned to previous or intended use. Horses with an unsuccessful outcome had advanced damage to the associated articular soft tissue structures and/or prolonged duration of lameness. Both horses with persistent fragments in the joint (Cases 12 and 14) did not achieve soundness. Therefore, it may be advisable that arthroscopic removal of the MICET fragments be performed early after diagnosis to minimise extent and severity of concomitant damage to articular soft tissue structures, which have a direct effect on prognosis for horses suffering from stifle conditions 3, 18.

Study limitations include the small number of cases precluding statistical analysis and its retrospective design whereby some information is not available. Inclusion of different hospitals and surgeons and subjective individual assessments may have been a source of variability in the data.

Fractures of the MICET occur in horses of different ages, are associated with a traumatic event in most of the cases and can be accompanied by a variable degree of soft tissue damage. This study provides further evidence that these fractures are not avulsion fractures of the CraCL, although the CraCL may be involved because of its anatomical proximity. Complete recovery may be expected in cases with mild concurrent lesions. Prompt diagnosis and arthroscopic removal of the fractured MICET are recommended as longer standing cases are associated with more severe articular soft tissue lesions and, therefore, a less favourable prognosis.

## Authors' declaration of interests

No competing interests have been declared.

## Ethical animal research

This study underwent ethical review by the Veterinary Research Ethics Committee of the Institute of Veterinary Science University of Liverpool and was granted ethical approval on 22.01.2014 (reference VREC276). Explicit owner informed consent for inclusion of animals in this study was not stated.

## Source of funding

None.

## Authorship

All authors were involved in study conception, design, contribution of cases and production of the manuscript. Luis M. Rubio‐Martínez conceived the idea for and led the study.

## Supporting information


**Supplementary Item 1:** Case background and clinical examination.

**Supplementary Item 2:** Intra‐articular anaesthesia and diagnostic imaging.

**Supplementary Item 3:** Intraoperative arthroscopic views of the axial aspect of the medial femorotibial joint of horses with fracture of the MICET.

**Supplementary Item 4:** Arthroscopic approach and findings.

**Supplementary Item 5:** Convalescence time and outcome.
