# Supplementary material for: Fracture of the medial intercondylar eminence of the tibia in horses treated by arthroscopic fragment removal (21 horses)
Source: Equine Vet J. 2017 Aug 15;50(1):60–4. doi: 10.1111/evj.12720 (PMC5724496; doi:10.1111/evj.12720)
Supplement: Supplementary file 1 — Supplementary Item 1: Case background and clinical examination. [file EVJ-50-60-s001.pdf]

Equine Veterinary Journal

Supplementary Item 1: Signalment and clinical examination.

**Case**	**Age**	**Sex**	**Breed**	**Use**	**Subjective lameness grade**	**Duration of lameness (days)**
**1**	10	G	TB	XC	3/10	90
**2**	18	G	Cob	General	4/10	U
**3**	4	F	Cob	Riding club	3/10	63
**4**	14	F	ISH	Retired	4/10	U
**5**	11	M	PBS	D	4/5	3
**6**	7	G	SF	SJ	1/5	42
**7**	12	F	Mixed	D	2/5	U
**8**	12	G	Polo	Polo	5/10	70
**9**	4	G	TB	Racing	Not recorded	U
**10**	11	G	Cob	General	2/10	U
**11**	14	G	Arab X	Unknown	6/10	28
**12**	8	F	Morgan	Broodmare	4/5	35
**13**	1	G	QH	Western	4/5	21
**14**	8	F	AP	Reining	4/5	4
**15**	8	F	W X	General	3/10	U
**16**	17	F	W	Riding Club	3/5	3
**17**	10	F	WB X	Dressage	4/5	U
**18**	10	G	OB	SJ	1/5	U
**19**	8	G	WB X	SJ	3/5	U
**20**	10	G	WB	Hunting	2/5	60
**21**	10	G	KWPN	D	5/5	1

Signalment and clinical examination findings on 21 horses with fracture of the medial intercondylar eminence of the tibia (MICET) and included in the study (AP = Appaloosa; D = Dressage; F = Female; G = Gelding; ISH = Irish Sport Horse; M = Male; OB = Oldenburger; PBS = Purebred Spanish; Polo = Polo pony; QH = Quarter Horse; SF = Selle Francais; SJ = Showjumping; TB = Thoroughbred; U = Unknown; W = Welsh; WB = Warmblood; X = cross; XC = Cross country).
