# Supplementary material for: Fracture of the medial intercondylar eminence of the tibia in horses treated by arthroscopic fragment removal (21 horses)
Source: Equine Vet J. 2017 Aug 15;50(1):60–4. doi: 10.1111/evj.12720 (PMC5724496; doi:10.1111/evj.12720)
Supplement: Supplementary file 2 — Supplementary Item 2: Intra‐articular anaesthesia and diagnostic imaging. [file EVJ-50-60-s002.pdf]

Equine Veterinary Journal

Supplementary Item 2: Intra-articular anaesthesia and diagnostic imaging.

Case	Improvement after IAA	RAD projections	MICET FX visualisation on RAD	Fragment quantity	Fragment size (mm)	Other RAD findings	MICET FX visualization on USE	Soft tissue damage on USE	USE equipment
**1**	40%	CdCr, LM, CdL-CrMO, flexed LM	Yes	1	22x7	No	Yes	No	1a
**2**	>50%	CdCr, LM, CdL-CrMO, flexed LM	Yes	1	13x7	No	Yes	Medial prolapse and mixed echogenicity of MM	1a
**3**	N/A	CdCr, LM, CdL-CrMO, flexed LM	Yes	3	26x8; 7x2; 4x3	Focal mineralisation of MM, osteophystosis	Yes	Mixed echogenicity of MM	1a
**4**	>50%	CdCr, LM, CdL-CrMO	Yes	4	37x13; 3 fragments of 4-6mm diameter	Osteophytosis, MFTJ collapse	Yes	Mixed echogenicity and irregular margins of MM	1a
**5**	N/A	CdCr, LM	Yes	1	15x22	No	No	No	Unknown
**6**	N/A	CdCr, LM, Flexed LM	Yes	1	30x10	No	No	No	Unknown
**7**	N/A	CdCr, LM	Yes	1	17x13	No	N/A	N/A	N/A
**8**	N/A	CdCr, Flexed LM	Yes	3	6x20	Flattening MFC	N/A	N/A	N/A
**9**	N/A	CdCr, LM, CdL-CrMO	Yes	1	Unavailable	No	N/A	N/A	N/A
**10**	>50%	CdCr, LM, CdL-CrMO, Flexed LM	Yes	1	5x15mm	No	N/A	N/A	N/A

**11**	>50%	CdCr, LM, Flexed LM	Yes	1	5x8x18	No	N/A	N/A	N/A
**12**	N/A	CdCr, LM, Flexed LM	Yes	>6	2-15 mm in diameter	Osteophytosis	N/A	N/A	N/A
**13**	N/A	CdCr, LM, Flexed LM	Yes	2	Unavailable	No	No	No	3a
**14**	N/A	CdCr, LM, Flexed LM	Yes	8	2-15 mm in diameter	No	Yes	Disruption of medial CL of MFTJ; medial prolapse of MM	3a
**15**	>50%	CdCr, LM, Flexed LM	Yes	1	20x5x7	Osteophytosis	Yes	No	1a
**16**	>50%	CdCr, LM, CdL-CrMO	No (poor quality)	1	10x20	None (poor quality radiographs)	No	No	1a
**17**	N/A	LM, CdCr, Flexed LM	Yes	1	30x12	No	No	Detachment MM from medial MFTJ CL.	2b
**18**	N/A	LM, CdCr, CdL-CrMO, Flexed LM	No (joint collapse)	1	20x10	Osteophytosis, MFTJ collapse	Yes	Medial MM prolapse; irregular tibial attachment of CraMTL	2b
**19**	>50%	LM, CdCr, Flexed LM	Yes	3	13x8; 5x5; 13x16	No	Yes	No	2a
**20**	>50%	CdCr, LM, CdL-CrMO	Yes	1	30x10	Proximal avulsion fracture MPL	Yes	No	4a
**21**	N/A	CdCr, LM, CdL-CrMO	Yes	1	50x15	No	Yes	Core hypoechogenicity in proximal aspect MPL	4a

Results of intra-articular anaesthesia and radiographic and ultrasonographic examinations on 21 horses with fracture of the medial intercondylar eminence of the tibia (MICET) and included in this study. The ultrasound equipment used was: [1]LOGIQe GE 12L-RS 4.0-13.0 MHz1; [2]ESAOTE

MyLab Class C, LA523 (7.5-10.0 MHz); [3]unknown machine, 8-14.0 MHz; [4]unknown machine, 7-12 MHz; [a]Linear and micro-convex probes; [b]Linear probe only (CdCr = Caudocranial; CdL-CrMO = Caudolateral-craniomedial oblique; CL = Collateral ligament; Cra MTL = Cranial meniscotibial ligament; FX = Fracture; IAA = Intra-articular anaesthesia; LM = Lateromedial; MFC = Medial femoral condyle; MFTJ = Medial femorotibial joint; MM = Medial meniscus; MPL = Middle Patellar Ligament; N/A = Not applicable as either IAA or USE was not performed for that case; RAD = Radiographic examination; USE = Ultrasonographic examination; *:other than synovial effusion and synovial membrane thickening). Fragment size in cases 16 and 18 was determined at surgery.
