# Supplementary material for: Fracture of the medial intercondylar eminence of the tibia in horses treated by arthroscopic fragment removal (21 horses)
Source: Equine Vet J. 2017 Aug 15;50(1):60–4. doi: 10.1111/evj.12720 (PMC5724496; doi:10.1111/evj.12720)
Supplement: Supplementary file 3 — Supplementary Item 3: Intraoperative arthroscopic views of the axial aspect of the medial femorotibial joint of horses with fracture of the MICET. [file EVJ-50-60-s003.pdf]

Equine Veterinary Journal

Supplementary Item 3: Intra-operative arthroscopic views of the axial aspect of the medial femorotibial joint of horses with fracture of the MICET.

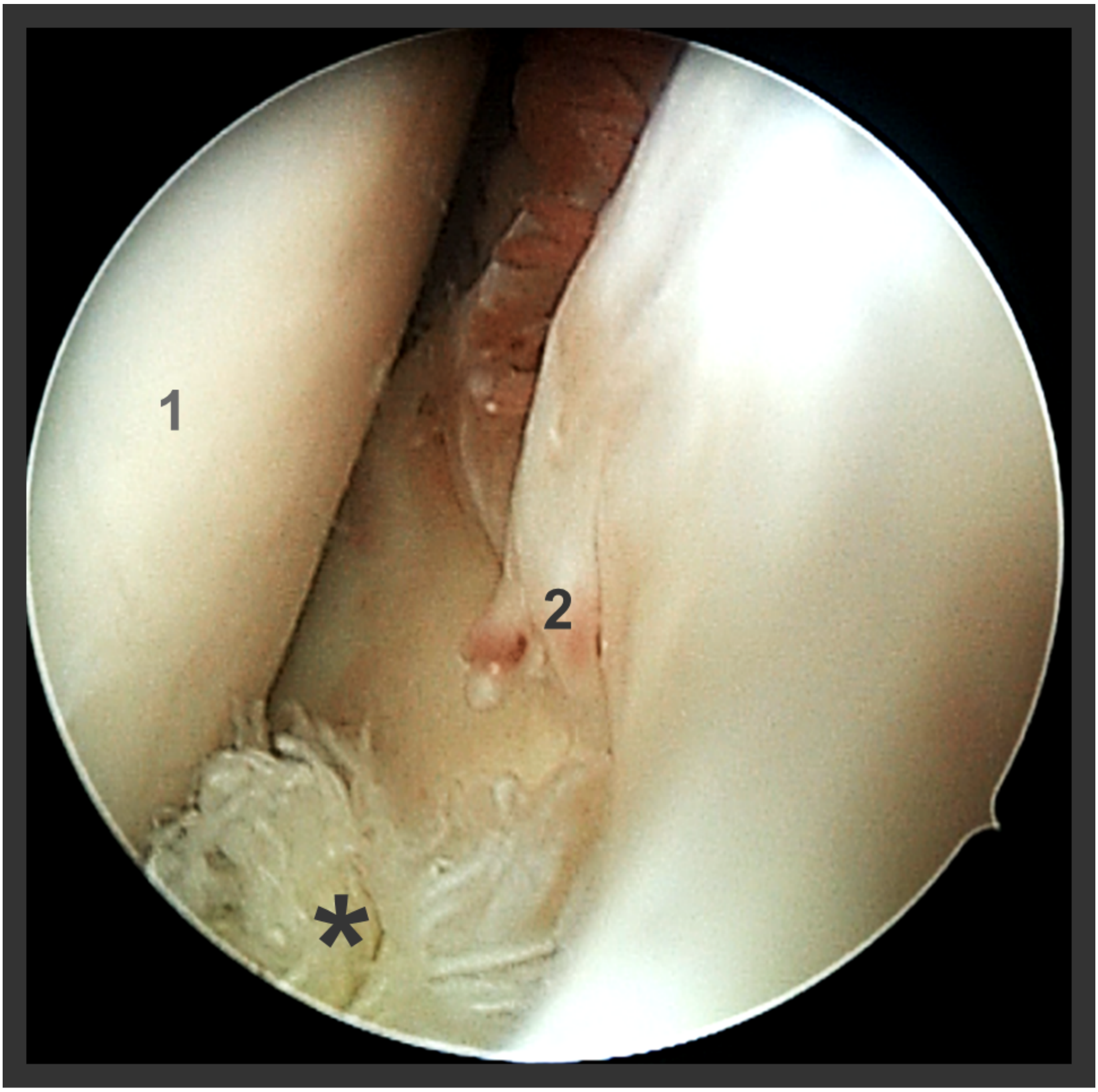

Medial is to the left of the image; proximal is to the top of the image. In A, a partial tear to the cranial ligament of the medial meniscus is evident (*). In B, a fragment from the MICET is elevated with a curette (1: medial femoral condyle; 2: fractured MICET).

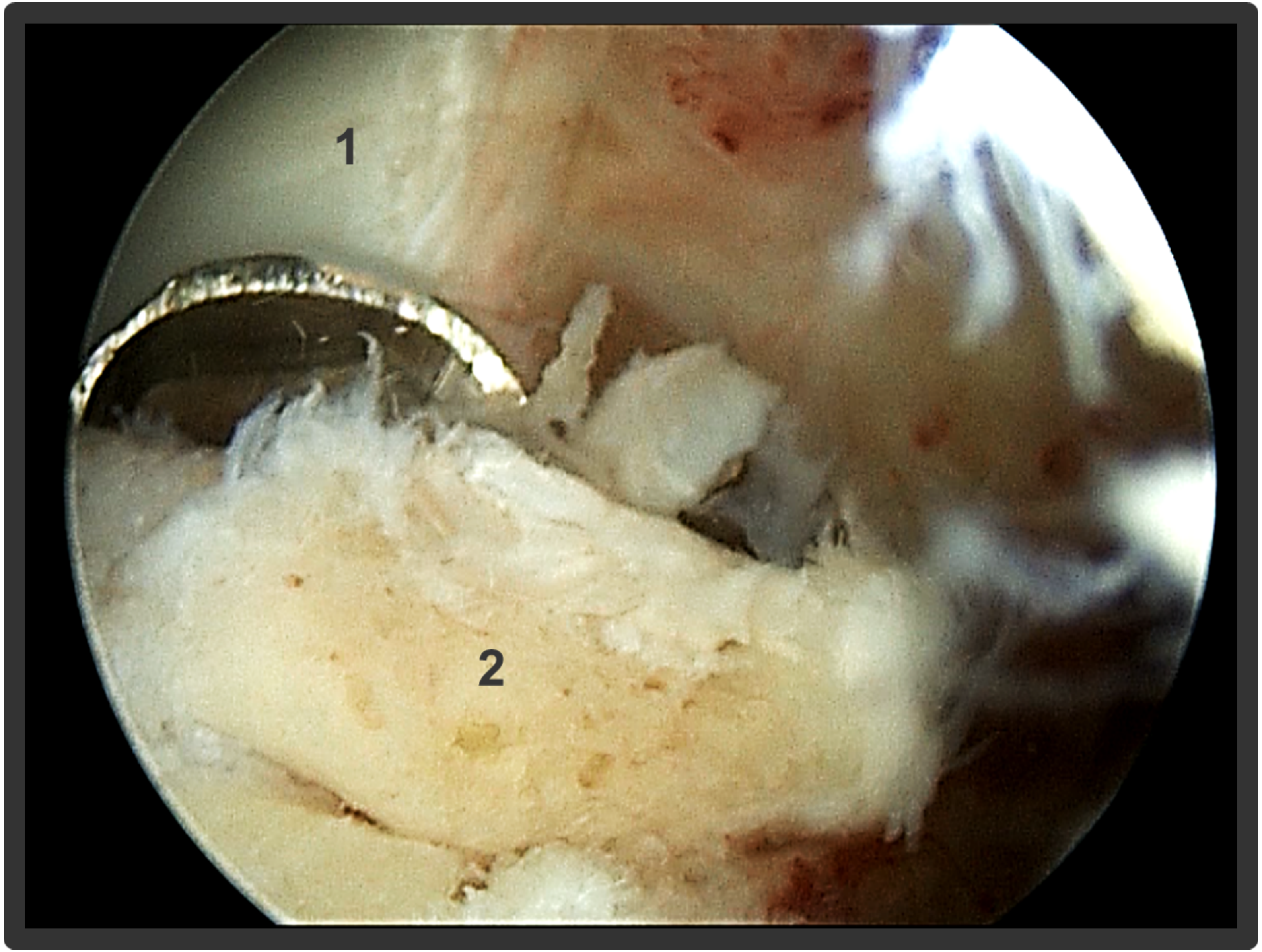
1
2
