# Supplementary material for: Fracture of the medial intercondylar eminence of the tibia in horses treated by arthroscopic fragment removal (21 horses)
Source: Equine Vet J. 2017 Aug 15;50(1):60–4. doi: 10.1111/evj.12720 (PMC5724496; doi:10.1111/evj.12720)
Supplement: Supplementary file 4 — Supplementary Item 4: Arthroscopic approach and findings. [file EVJ-50-60-s004.pdf]

Equine Veterinary Journal

Supplementary Item 4: Arthroscopic approach and findings.

Case	Arthroscopic approach MFTJ	Cartilage damage MFC	Damage CraCL	Damage MM	Damage cranial ligament of MM	Arthroscopic findings FPJ	Arthroscopic findings LFTJ
1	Lateral	Intact	0%	Intact	0%	N/A	No
2	Cranial	Intact	10%	Intact	20%	N/A	Mild cartilage degeneration
3	Cranial after failed lateral	Moderate	30%	Tear grade 2	40%	N/A	No
4	From FPJ	Moderate	60%	Tear grade 3	35%	No	Mild cartilage degeneration
5	From FPJ	Mild	25%	Intact	0%	No	No
6	Lateral	Mild	50%	Intact	25%	N/A	No
7	From FPJ	Mild	25%	Intact	25%	No	N/A
8	Lateral	Mild	50%	Intact	10%	N/A	No
9	Lateral	Intact	25%	Intact	0%	N/A	N/A
10	Lateral	Moderate	10%	Intact	5%	N/A	Mild damage to CraMTL
11	Lateral	Moderate	10%	Tear grade 1	0%	N/A	Mild damage to CraMTL
12	Lateral	Not recorded	50%	Not recorded	0%	N/A	Mild damage to CraMTL
13	Lateral	Intact	0%	Intact	0%	N/A	No
14	Lateral	Mild	60%	*	50%	N/A	No
15	Cranial	Moderate	0%	Intact	0%	No	Mild cartilage degeneration
16	Lateral	Mild	0%	Intact	0%	N/A	No

**17**	Lateral	Intact	0%	Intact	10%	N/A	No
**18**	Lateral	Moderate	0%	Intact	0%	N/A	No
**19**	Lateral	Intact	10%	Intact	0%	N/A	No
**20**	From FPJ	Mild	15%	Intact	5%	N/A	No
**21**	From FPJ	Mild	25%	Tear grade 1	15%	N/A	No

Arthroscopic examinations performed and significant findings observed in the medial and lateral medial femorotibial and femoropatellar joints on the 21 horses with fracture of the medial intercondylar eminence of the tibia (MICET) and included in this study. The arthroscopic approaches to the MFTJ were performed as previously described [7]. Damage of articular cartilage was graded as mild (few superficial wear lines); moderate (deep but not full thickness wear lines or areas with partial thickness loss of articular cartilage) or severe (full thickness loss of articular cartilage and subchondral bone exposure). Percentages represent the estimated damage to the individual ligaments as % of cross-section area involved. Presence of meniscal tear is graded as previously reported [7] (CL = Collateral ligament; Cra MTL = Cranial meniscotibial ligament; FPJ = Femoropatellar joint; LFTJ = Lateral femorotibial joint; MFC = Medial femoral condyle; MFTJ = Medial femorotibial joint; N/A = Not applicable as arthroscopic examination of either FPJ or LFTJ was not performed for that case; *MM appeared intact but separated from medial CL).
