# Supplementary material for: Fracture of the medial intercondylar eminence of the tibia in horses treated by arthroscopic fragment removal (21 horses)
Source: Equine Vet J. 2017 Aug 15;50(1):60–4. doi: 10.1111/evj.12720 (PMC5724496; doi:10.1111/evj.12720)
Supplement: Supplementary file 5 — Supplementary Item 5: Convalescence time and outcome. [file EVJ-50-60-s005.pdf]

Equine Veterinary Journal

Supplementary Item 5: Convalescence time and outcome.

Case	Time of follow-up (months)	Follow-up examination performed by	Total convalescence time (months)	Return to previous use	Soundness achieved
1	9	Owner and RVS	5	Yes	Yes
2	18	Owner and RVS	8	Yes	Yes
3	N/A	Owner and RVS	Euthanised for persistent lameness	No	No
4	N/A		Euthanised 6 days after surgery for persistent severe lameness	No	No
5	10	AVS	7	Yes	Yes
6	42	AVS	7	Yes	Yes
7		AVS	In rehab	In rehab	Yes
8	27	RVS	9	No*	Yes
9	14	RVS and online racing records	8	Yes	Yes
10	66	RVS	6	Yes	Yes
11	N/A		9		
12	72	RVS	Unknown	Yes	No
13	36	RVS	Unknown	Yes	Yes
14	30	RVS	Unknown	No	No
15	27	RVS	Sustained injury whilst in rehab	Yes	Yes
16	12	AVS	6	Yes	Yes
17	14	RVS	5	Yes	Yes
18	10	AVS	5	Yes	Yes
19	4	AVS	6	In rehab	Yes

20	12	AVS	4	Yes	Yes
21	4	AVS	4	In rehab	Yes

Individual details on follow-up information, convalescence time and outcome on 21 horses with fracture of the medial intercondylar eminence of the tibia (MICET) included in the study (AVS = Attending Veterinary Surgeon; HA = Hyaluronic acid; Rehab = Rehabilitation; IA = Intra-articular; IRAP = Interleukin-receptor antagonist; N/A = Not applicable as follow-up was not available or horse had been euthanised for persistent lameness; RVS = Referring Veterinary Surgeon; * = owners elected general riding use).
